# Absence of Toll-like Receptor 21 (*TLR21*) Gene in the Genome of Transparent Glass Catfish (*Kryptopterus vitreolus*)

**DOI:** 10.3390/biology15030263

**Published:** 2026-02-01

**Authors:** Shengtao Guo, Xinhui Zhang, Rusong Zhang, Kai Zhang, Jianchao Chen, Yunyun Lv, Zhengyong Wen, Jieming Chen, Chao Bian, Qiong Shi

**Affiliations:** 1 Laboratory of Aquatic Genomics, College of Life Sciences and Oceanography, Shenzhen University, Shenzhen 518057, China; guoshengtao18@mails.ucas.ac.cn (S.G.); zhangxhui1987@163.com (X.Z.); 15940336815@163.com (R.Z.); zhangkai1@szu.edu.cn (K.Z.); 2410173010@mails.szu.edu.cn (J.C.); 2 Fishes Conservation and Utilization in the Upper Reaches of the Yangtze River Key Laboratory of Sichuan Province, College of Fisheries, Neijiang Normal University, Neijiang 641100, China; lvyunyun_sci@foxmail.com (Y.L.), zhengyong_wen@126.com (Z.W.); 3 Center for Aquatic Genomics, BGI Academy of Marine Sciences, Shenzhen 518081, China; chen_jm02@163.com

**Keywords:** glass catfish, Toll-like receptor 21 (*TLR21*), comparative genomics, immune adaptation

## Abstract

This study investigates the genomic basis of immune adaptation in the transparent glass catfish (*Kv*: *Kryptopterus vitreolus*), focusing on the loss of the Toll-like receptor 21 (*TLR21*) gene. Comparative genomic analysis with closely related non-transparent North African catfish (*Cg*: *Clarias gariepinus*) revealed 11 *TLR* genes in the latter, while only 8 *TLR* genes (*KvTLR1*, *2*, *3*, *5*, *7*, *9*, *13*, and *20*) were retained in the glass catfish, with *TLR21* specifically absent. Collinearity analysis confirmed that the genomic region containing *TLR21* is conserved across eight siluriform species, with loss exclusively in the glass catfish, supporting its lineage-specific absence. Structural expansion was notable in *KvTLR5*, *KvTLR7*, and *KvTLR20*. Molecular docking indicated that binding stability between CpG oligonucleotides and TLR21 varies significantly, with CpG-B 1681 showing the strongest interaction, which highlights sequence-dependent ligand recognition. Interestingly, absence of the *TLR1* gene in another transparent teleost, the X-ray tetra (*Pristella maxillaris*), suggests that transparent fishes may share an evolutionary trend of lineage-specific *TLR* gene loss. Together, these findings reveal a distinctive evolutionary trajectory in the innate immune receptor family of transparent fishes and provide new molecular insights into their adaptive immune strategies. These insights will benefit the academic community by improving comparative frameworks for fish innate immunity, and they may inform disease prevention and health management strategies in aquaculture and the ornamental fish trade.

## Simple Summary

Transparent fishes, such as the ornamental glass catfish, have evolved unique transparent bodies, but how their immune system adapts for defense against exogenous pathogens remains unclear. This study compared the genomes of the glass catfish and its close relative, the non-transparent North African catfish. We found that the glass catfish has lost an important immune gene called *TLR21*, which is present in most other fish species. Meanwhile, some of the remaining immune genes in the glass catfish have been expanded to become more complex. Our results showed that the TLR21 protein in the North African catfish can potentially recognize pathogen molecules, but its binding strength varies greatly depending on the molecular sequence from various teleosts. We also discovered that another transparent fish, the X-ray tetra, has lost another *TLR* gene, *TLR1*. These interesting findings suggest that transparent fishes may have adjusted their immune defenses during evolution by losing certain immune gene(s) while strengthening others. In summary, this research provides new insights into how transparent fishes adapt to the aquatic environment, and improves our understanding of the immune evolution in fish.

## 1. Introduction

The immune system of transparent fish may possess unique characteristics due to their distinct physiological changes. For example, their transparent body walls are closely exposed to external pathogens, suggesting potential adaptive evolutionary features in their immune defense mechanisms. However, the composition and function of the immune system in representative glass catfish (*Kv*: *Kryptopterus vitreolus*), an economically important transparent fish species, remain unstudied to date, although we have recently reported its chromosome-level genome assembly [1]. Our current research also selects its close relative, non-transparent North African catfish (*Cg*: *Clarias gariepinus*) [2], as the study subject. The two species are phylogenetically closely related within the same order Siluriformes, and the immune genes of *C. gariepinus* have been well annotated [3]. Through comparative genomics and molecular examinations, our current study aims to provide new insights into the immune adaptation mechanisms of the glass catfish and, more broadly, transparent fish species.

Fish immune system usually serves as a critical transitional model between innate and adaptive immunity in vertebrates, relying heavily on pattern recognition receptors for rapid detection of pathogen-associated molecular patterns [4,5]. Among these, the Toll-like receptor (TLR) family has undergone significant gene expansion and functional diversification in fish [6], encompassing diverse subtypes, such as TLR1, 2, 3, 5, 7/8/9, and 21 [7,8]. These receptors recognize conserved molecular structures of bacteria, viruses, fungi, and other pathogens, activating signaling pathways such as NF-κB or interferon regulatory factors to initiate the expression of inflammatory cytokines and antiviral proteins [9]. As the first molecular line of defense against exogenous pathogen invasion in fish, TLRs have become a key focal point for studying immune evolution in vertebrates and host-pathogen interactions [10].

TLR21 is an important member of the TLR family [11], although research on its function has primarily focused on the recognition of pathogen-associated molecular patterns (PAMPs) and its involvement in innate immune responses [12]. Fish TLR21 has been confirmed to recognize unmethylated CpG DNA, a conserved feature of bacterial and viral DNA [13]. Unlike its functional homolog TLR9 in mammals, fish TLR21 can directly recognize CpG DNA in extracellular or endosomal compartments and activate the downstream MyD88-dependent signaling pathway, thereby inducing the production of type I interferons and pro-inflammatory cytokines [14] for immunity. In various fish species (such as common carp and yellowtail amberjack), *TLR21* expression is significantly upregulated following bacterial or viral infection [15,16]. *TLR21* is often highly expressed in immune-related tissues of fish, such as the spleen, head kidney, and gills [17]. Its protein structure includes a typical leucine-rich repeat (LRR) extracellular domain (for ligand binding), a transmembrane domain, and a Toll/interleukin-1 receptor (TIR) intracellular domain [18]. It is usually localized to endosomal membranes, facilitating contact with internalized pathogenic DNAs [19].

Evolutionarily, *TLR21* and *TLR9* likely originated from a common ancestral gene [20]. The presence of both *TLR9* and *TLR21* in zebrafish, which have distinct ligand specificity but act cooperatively in response to CpG-ODNs, illustrates the functional complexity and integration of the teleost TLR system [21]. As an important pattern recognition receptor, fish TLR21 always activates immune signaling pathways by recognizing pathogenic DNAs, playing a central role in defending against bacterial and viral infections [22,23].

Teleost TLR1 typically forms a heterodimer with TLR2 (TLR1/2 complex) to jointly recognize bacterial lipoproteins, lipopeptides, and other pathogen-associated molecules [24]. This complex activates nuclear factor kappa B (NF-κB) through the MyD88-dependent signaling pathway to induce the expression of pro-inflammatory cytokines and antimicrobial peptides, thereby playing a critical defensive role in mucosal barrier tissues such as gills and intestines [25]. Previous studies have shown that its expression is significantly upregulated upon bacterial infection, and loss of its function impairs pathogen clearance, highlighting its essential role in defending against exogenous pathogens (such as Gram-positive bacteria) within the teleost innate immune system [26,27]. In teleost, *TLR21* generally shows a broad tissue distribution but is typically enriched in immune-related organs, suggesting a primary role in host defense. For example, in yellow catfish, *TLR21* transcripts can be detected across multiple tissues (including spleen, head kidney, trunk kidney, liver, gill, and blood), with the highest expression in the spleen, and its expression profile is described as similar to that of *TLR9* [28]. Likewise, in golden pompano, *TLR21* is constitutively expressed in a panel of tissues (such as skin, gill, kidney, spleen, liver, and intestine) and it is highly expressed in the spleen and head kidney, further supporting its immunological relevance [29]. Consistent with this expression pattern, teleost *TLR21* is also responsive to pathogen challenge and immune stimulation, displaying induction patterns that are both tissue- and stimulus-dependent. In yellow catfish, *TLR21* mRNA was upregulated in spleen, head kidney, and other immune-relevant tissues following challenge with killed *Aeromonas hydrophila* [29]. In golden pompano, *TLR21* was markedly upregulated after exposure to bacterial infection (*Vibrio alginolyticus*) and classical PAMP (pathogen-associated molecular patterns) mimics such as LPS (lipopolysaccharide) and poly (I:C), with clear time-course dynamics in spleen and head kidney [28].

Our present study employs a comparative genomics approach, with focusing on the *TLR* gene family, to systematically compare the composition of *TLR* genes between the typical transparent glass catfish and its close relative, North African catfish. By analyzing potential lineage-specific expansions, functional differentiation, or losses within the *TLR* family of the glass catfish, this research aims to reveal the molecular mechanisms underlying environmental adaptation in its immune system at the pattern recognition receptor level. Furthermore, we seek to provide evidence to elucidate the evolutionary relationship between the unique physiological structure of transparent fish and their immune defense strategies. This study thereby aims to uncover evolutionary signatures of immune adaptation in transparent fishes, which may provide new insights into the adaptive evolution of vertebrate immune gene families and help establish molecular connections between physiological specialization and immune adaptation in teleosts.

## 2. Materials and Methods

### 2.1. Fish Species, Genome Data, and Gene Nomenclature

In this study, the transparent glass catfish *Kryptopterus vitreolus* (*Kv*) was used as the central species for study. Its closely related non-transparent species, the North African catfish *Clarias gariepinus* (*Cg*), was selected for genomics comparison. For collinearity analysis of *TLR21*, eight Siluriformes species were chosen, including *Clarias fuscus* (Hong Kong catfish), *C. gariepinus* (North African catfish), *K. vitreolus* (glass catfish), *Ictalurus furcatus* (blue catfish), *Pangasianodon hypophthalmus* (striped catfish), *Pelteobagrus vachelli* (Vachelli catfish), *Tachysurus fulvidraco* (yellow catfish), and *Trichomycterus rosablanca* (Andean caved catfish). For the collinearity analysis of *TLR1*, another transparent fish, the X-ray tetra (*Pristella maxillaris*) was further included, together with six non-transparent teleost species (*C. gariepinus*, *P. fulvidraco*, *P. hypophthalmus*, *P. vachelli*, *I. furcatus*, and *T. rosablanca*).

Chromosome-level reference genome assemblies of the above-mentioned species were downloaded from public databases such as NCBI and/or Ensembl (Table 1), along with their corresponding genome annotation files (GFF/GTF), coding sequences (CDS), and predicted protein datasets.

### 2.2. Genome-Wide Identification of TLR Genes

To identify *TLR* genes in the genomes of *Kv* and *Cg*, protein sequences of previously reported *TLR*s (including *TLR1*, *2*, *3*, *5*, *7*, *8*, *9*, *13*, *18*, *20*, and *21*) were first collected from multiple teleost species as the query sequences. BLASTP (v2.2.6; NCBI, Bethesda, MD, USA) [30] and TBLASTN (v2.2.6; Bethesda, MD, USA, NCBI) searches were then performed against the protein and genome databases of *Kv* and *Cg*, with an E-value threshold of 1 × 10^−5^ [31]. Redundant hits and partially overlapping sequences from the genomes were manually inspected and merged according to their genomic coordinates.

Subsequently, SMART 7 [32], Pfam (EMBL-EBI, Hinxton, Cambridgeshire, UK) [33], and InterPro (v101.0; EMBL-EBI, Hinxton, Cambridgeshire, UK) [34] were employed to predict and confirm the domain architecture of candidate TLR proteins, with particular attention to whether they contained the typical Toll-like receptor domains, namely an extracellular LRR region (a single transmembrane domain) and an intracellular TIR domain. Sequences lacking a TIR domain or with severely incomplete LRR structures were considered as non-canonical or truncated for removal from the final gene list.

For each confirmed *TLR* gene, its genomic length, CDS length, open reading frame (ORF), predicted amino acid number, and theoretical molecular weight were calculated based on the coding sequence. Genomic positions (chromosome or scaffold IDs and start–end coordinates) were obtained from the genome annotation files or determined by aligning the CDS to the target genome using BLASTN (v2.2.6; NCBI, Bethesda, MD, USA) [35].

### 2.3. Phylogenetic Analysis and Domain Composition of TLRs in Clarias gariepinus

To analyze the evolutionary relationships within the TLR family of *Cg*, all identified *Cg*TLR protein sequences (*Cg*TLR1, 2, 3, 5, 7, 8, 9, 13, 18, 20, and 21) were aligned using MAFFT version 5 with default parameters [36]. Regions with poor alignment quality or extensive gaps were manually trimmed to reduce noise for phylogenetic reconstruction.

In a phylogenetic analysis, the bootstrap value is used to evaluate the statistical reliability of branching patterns. A phylogenetic tree was then constructed using IQ-TREE (v3; IQ-TREE team, Vienna, Austria) based on the maximum likelihood (ML) method [37]. The best-fit amino acid substitution model was automatically selected using ModelFinder (implemented in IQ-TREE v3; developed by the IQ-TREE team, Vienna, Austria) [38], and node supports were evaluated with 1000 bootstrap replicates. In this study, the reliability of key evolutionary branches was further validated using Bayesian inference [39].

### 2.4. Collinearity Analysis of TLR21 in Siluriformes and Other Teleost Species

To examine the conservation and loss patterns of *TLR21* in Siluriformes, the genomic region containing *TLR21* was first identified in the *Cg* genome, and ten flanking genes on both sides (including *USP5*, *CD4*, *CXCR3*, *CXCR3_2*, *CXCR3_1*, *CNFN*, *PRR19*, *PAFAH1B3*, and *CEACAM*) were selected to construct a reference syntenic block. This block was then searched in the genomes of eight Siluriformes species (including *Clarias fuscus*, *Cg*, *Kv*, *I. furcatus*, *P. hypophthalmus*, *P. vachelli*, *T. fulvidraco*, *T. rosablanca*) using BLASTP (v2.2.6; NCBI, Bethesda, MD, USA) with an E-value < 1 × 10^−10^ to identify orthologous genes.

The gene order, transcriptional orientation, and intergenic distances within this syntenic block were compared across species, and collinear blocks were identified and visualized using TBtools II (v0.665) [40]. The status (presence or absence) of *TLR21* in each species was confirmed by examining both annotated genes and unannotated genomic sequences.

To further characterize the evolutionary pattern of *TLR21*, its coding sequences were collected from representative species within four teleost orders: Perciformes (yellowfin tuna *Thunnus albacares*, Southern bluefin tuna *Thunnus maccoyii*, turbot *Scophthalmus maximus*), Pleuronectiformes (European flounder *Platichthys flesus*, European plaice *Pleuronectes platessa*), Siluriformes (*Cg*, *P. hypophthalmus*, *I. furcatus*), and Cypriniformes (*Cyprinus carpio*, goldfish *Carassius auratus*). Their deduced amino acid sequences were aligned, and an ML phylogenetic tree was constructed [37].

### 2.5. Molecular Docking Analysis of CgTLR21 with CpG ODN Molecules

A three-dimensional (3D) structure of the extracellular LRR region of *Cg*TLR21 was obtained by homology modeling using SWISS-MODEL (2018 version) [41], with structurally related TLR proteins as templates, or by using an available predicted model.

Nine CpG oligodeoxynucleotides (CpG ODNs) commonly used in fish and mammalian studies were selected as the ligands, such as CpG-A 8954, CpG-B 1681, CpG-B 2006, CpG-B 2143, CpG-P 21426, CpG-P 23617. The nine CpG ODN sequences selected for this study have all been experimentally validated in teleosts or crustaceans for their immunomodulatory functions, including activation of TLR9/TLR21 signaling pathways, induction of immune gene expression, and enhancement of antibacterial and antiviral responses [28,42]. An initial 3D structure of each CpG ODN was built by using molecular modeling and energy-minimized under the AMBER force field implemented in Gaussian (2018 version) [43], followed by format conversion for docking. In detail, molecular docking was performed using AutoDock 4.2.6 [44]. A docking grid was defined around the predicted ligand-binding region of the *Cg*TLR21 LRR domain. Multiple docking runs were conducted for each CpG ODN, and molecular docking was performed with 100 independent runs for each ligand. The conformation with the lowest binding energy (kcal/mol) was selected as the representative binding mode. Binding energies were compared among different CpG ODNs to evaluate their relative binding stability with *Cg*TLR21.

### 2.6. Collinearity Analysis of TLR1 in the X-Ray Tetra

In a trial of genome-wide identification of *TLR* genes, we observed genomic loss of *TLR1* in the genome of the X-ray tetra (*Pm*: *Pristella maxillaris*). To further investigate the detailed loss of *TLR1* in *Pm*, we identified the *TLR1* locus and its flanking paralogous gene block, including *Ics10*, *f8*, *Scf16*, *B-klotho*, *Ubi-E2*, and *nar* in the reference *Cg* genome. The corresponding gene block was then searched in the genomes of *Kv*, *P. fulvidraco*, *P. hypophthalmus*, *P. vachelli*, *I. furcatus*, *T. rosablanca*, and *Pm* using BLAST-based (v2.2.6; NCBI, Bethesda, MD, USA) homology searches to identify orthologous genes [45].

## 3. Results

### 3.1. Genomics Comparison of the TLR Gene Family in Glass Catfish and North African Catfish: Lineage-Specific Gene Loss and Structural Expansion

Through genomics comparison, this study reveals significant differences in the *TLR* gene family between the glass catfish (*Kv*) and its close relative, the North African catfish (*Cg*) (see Table 2). Only eight *TLR* genes, including *KvTLR1*, *2*, *3*, *5*, *7*, *9*, *13*, and *20*, were identified in the glass catfish, with absence of homologs for at least *TLR21*. Furthermore, the coding sequence lengths and predicted protein molecular weights of some *TLR* genes (such as *KvTLR5*, *TLR7*, and *TLR20*) in the glass catfish are significantly larger than those of their orthologs in the North African catfish. These findings suggest that the *TLR* family in transparent fish may have undergone an evolutionary process, characterized by lineage-specific *TLR* gene loss, coupled with structural domain expansion.

Subsequent genomic analysis identified a total of 11 *TLR* genes in the North African catfish, including *CgTLR1*, *2*, *3*, *5*, *7*, *8*, *9*, *13*, *18*, *20*, and *21*. These genes exhibit considerable variation in genomic length (ranging from 3199 to 9885 bp) and ORF size (from 2391 to 3201 bp), encoding predicted proteins of 797 to 1067 amino acids with molecular weights ranging from 92.4 to 123.3 kDa (see more details in Table 2). Correspondingly in the genome of the glass catfish, the eight *TLR* genes (*Kv*TLR1, 2, 3, 5, 7, 9, 13, and 20) also exhibit extensive variation in genomic length (ranging from 2882 to 32,561 bp) and ORF size (ranging from 1569 to 8319 bp; Table 2). Notably, the ORFs of *KvTLR5*, *7*, and *20* are significantly extended, suggesting potential evolutionary events of domain expansion or gene fusion.

### 3.2. Phylogeny of TLR Proteins in Clarias gariepinus: Distinct Evolutionary Status and Structural Conservation of TLR21

**Figure 1:**
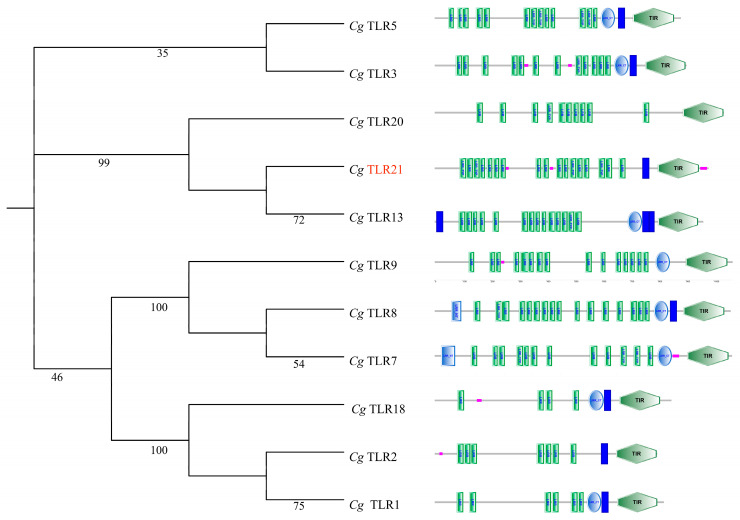
Phylogenetic tree (**left**) and domain architecture (**right**) of TLRs from *Clarias gariepinus* (*Cg*). These proteins, encoded by various *TLR* genes, consist of multiple domains, including a signal peptide, an extracellular LRR region (including LRR-N-terminal, central LRR, and LRR-C-terminal segments), a transmembrane (TM) domain, and a TIR domain. In the right panel, the TM domain is shown in dark blue, low-complexity regions are highlighted in pink, and other domains, such as LRR and TIR, are labeled separately.

The phylogenetic relationships of various TLR proteins from *Cg* are summarized in Figure 1, with bootstrap values for the support of each clade. *Cg*TLR21 is placed as a distinct protein, supported by a bootstrap value of 99. Other TLR proteins, such as *Cg*TLR5, 3, 20, 13, 9, 8, 7, 18, 2, and 1, form separate branches, with bootstrap values ranging from 35 to 100. *Cg*TLR21 is highlighted in orange to emphasize its importance for this study.

Additionally, the domain structures of these TLR proteins are displayed next to the phylogenetic tree (Figure 1), revealing conserved LRR and TIR domains across these TLRs, although some variations in domain organization are noted, particularly in those with low bootstrap support. Our results highlight TLR21 as a distinct and well-supported member of the TLR family, but its domain structure is generally consistent with the other TLRs.

### 3.3. Absence of TLR21 in the Glass Catfish Genome: Validated by Genomic Collinerity

**Figure 2:**
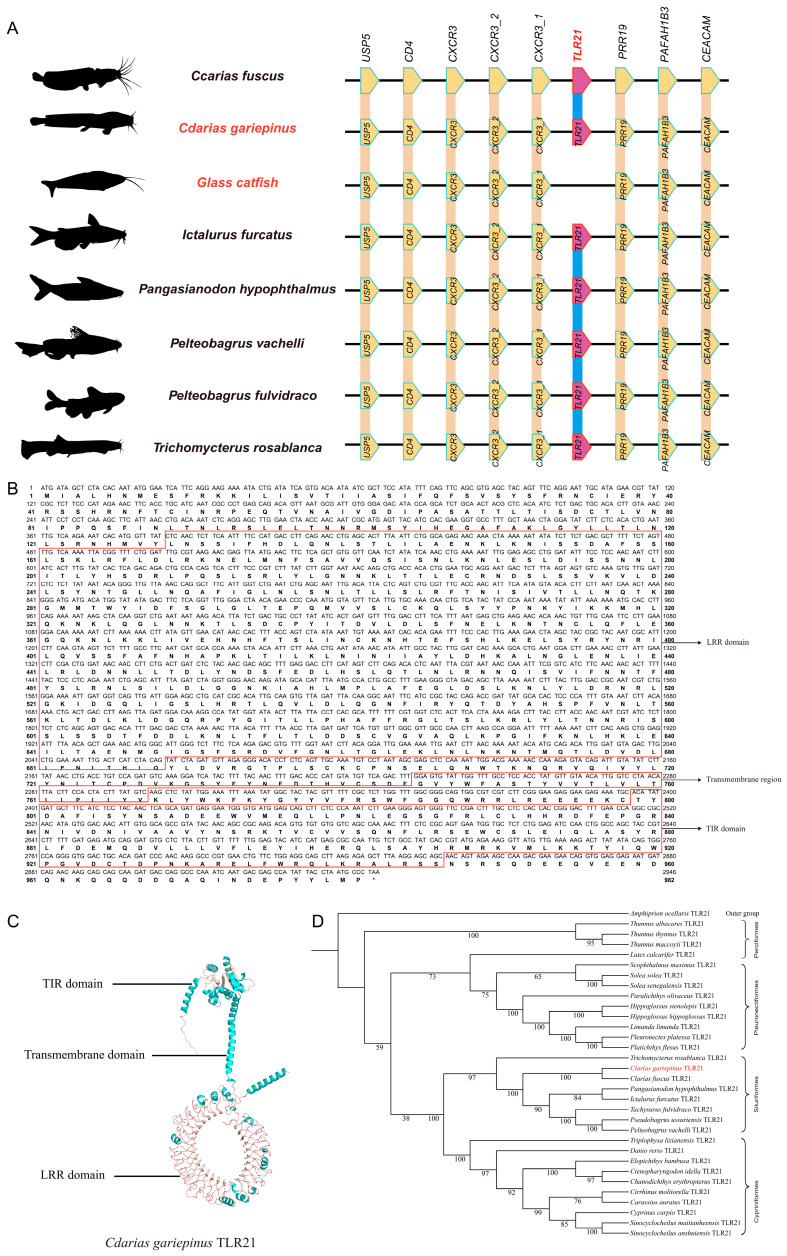
Absence of *TLR21* in the glass catfish genome: validated by genomic collinearity. (**A**) Conserved synteny surrounding *TLR21* across representative fish genomes, showing adjacent genes and the relative position of *TLR21*. (**B**) Nucleotide sequence and deduced amino acid sequence of *CgTLR21*, with annotated LRR domain, transmembrane region, and TIR domain. (**C**) Predicted protein structure of *Cg*TLR21, highlighting the LRR, transmembrane, and TIR regions. (**D**) Phylogenetic analysis of fish TLR21 proteins, showing the four major clades (Perciformes, Pleuronectiformes, Siluriformes, Cypriniformes) with high bootstrap support values. *Amphiprion ocellaris* (common clownfish) is used as the outgroup. The *TLR21* from the glass catfish is not included in this phylogenetic tree due to its absence in the genome, and therefore no corresponding TLR21 branch is present for comparison. “*” indicates a stop codon.

Having identified the *TLR* gene repertoire in the target genomes, we next examined syntenic conservation to validate the presence or absence of *TLR21* at the corresponding locus. We systematically examined ten neighboring genes (including *USP5*, *CD4*, *CXCR3*, *CXCR3_2*, *CXCR3_1*, *CNFN*, *TLR21*, *PRR19*, *PAFAH1B3*, and *CEACAM*) across eight siluriform species (Figure 2A). Our results demonstrate that the *TLR21* gene is consistently present in all examined species except the glass catfish. That is to say, other seven species retain an intact *TLR21* gene, while the glass catfish is the only one lacking this gene. The nine neighboring genes as the controls were detected with complete sequences in all the eight species, forming a distinct “present-in-all-absent-in-one” distribution pattern.

The complete coding sequence of the *CgTLR21* gene and the domain architecture of its encoded protein are shown in Figure 2B. As expected, this TLR protein contains three characteristic domains, including an extracellular LRR domain and an intracellular TIR domain (Figure 2C).

After the synteny evidence supported the lineage specific loss of TLR21 in the glass catfish, we further conducted phylogenetic analyses to resolve the phylogenetic relationships of TLR21 across teleost species. In the phylogenetic tree (Figure 2D), evolutionary relationships of the *TLR21* gene across fish species (without *Kv*) demonstrate its high conservation. These species in the tree are grouped into four major orders: Perciformes, Pleuronectiformes, Siluriformes, and Cypriniformes. Within these orders, the *TLR21* gene exhibits high conservative phylogeny across the examined species (without the glass catfish). The sampled fishes in Perciformes (*Thunnus albacares*, *Thunnus maccoyii* and *Scophthalmus maximus*), as well as those in Pleuronectiformes (representative *Platichthys flesus* and *Pleuronectes platessa*), all show similar *TLR21* nucleotide sequences. In Siluriformes, *Cg* forms a branch with other species like *Pangasianodon hypophthalmus* and *Ictalurus furcatus*, indicating a high level of the *TLR21* gene homology. In Cypriniformes, selected species such as *Cyprinus carpio* and *Carassius auratus* also show a high degree of conservation in the *TLR21* gene. Overall, the evolution of the *TLR21* gene in various fishes is highly conserved, while it is absent in the genome of the transparent glass catfish.

### 3.4. Binding Stability Differences Between CgTLR21 and Various CpG ODN Molecules for the Influence of Sequence Specificity

**Figure 3:**
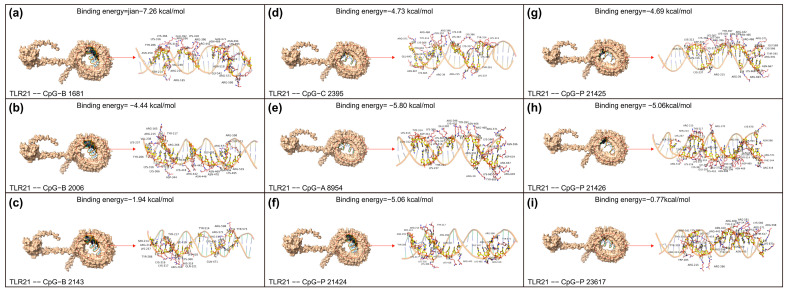
Group docking of *Cg*TLR21 with the common CpG ODNs from representative fishes and mammals. Docking poses are shown as a 3D surface view of the TLR21–CpG complex (left) and the corresponding 2D interaction map (right), with predicted binding energies (kcal/mol) indicated above each panel. (**a**) TLR21–CpG-B 1681 (−7.26). (**b**) TLR21–CpG-B 2006 (−4.44). (**c**) TLR21–CpG-B 2143 (−1.94). (**d**) TLR21–CpG-C 2395 (−4.73). (**e**) TLR21–CpG-A 8954 (−5.80). (**f**) TLR21–CpG-P 21,424 (−5.06). (**g**) TLR21–CpG-P 21,425 (−4.69). (**h**) TLR21–CpG-P 21,426 (−5.06). (**i**) TLR21–CpG-P 23,617 (−0.77).

With synteny analyses clarifying the conserved genomic context and the loss pattern, we then performed molecular docking to evaluate potential differences in binding between CgTLR21 and various CpG ODNs. Molecular docking revealed significant differences in binding stability among nine distinct types of CpG ODN with the *Cg*TLR21 receptor (Figure 3). Binding energy data showed a continuous distribution in interaction strength between the ligands and the target receptor. Among them, CpG-B 1681 demonstrated the strongest binding capacity (binding energy: −7.26 kcal/mol), with its absolute value significantly higher than other molecules, suggesting that this complex may form the most stable binding conformation. Meanwhile, CpG-A 8954 (−5.80 kcal/mol) and CpG-P 21,426 (−5.06 kcal/mol) exhibited moderately high binding stability.

Notably, significant differences were also observed within the same type of CpG molecules. For instance, in the CpG-B series, the binding energy difference between no. 1681 and no. 2006 (−4.44 kcal/mol) or no. 2143 (−1.94 kcal/mol) reached 5.32 kcal/mol, indicating that sequence specificity may have a greater impact on binding stability than type differences. Particular attention should be paid to CpG-P 23,617 (−0.77 kcal/mol) and CpG-B 2143 (−1.94 kcal/mol), as their relatively high binding energy suggest potential unfavorable factors in their binding conformations with *Cg*TLR21, such as steric hindrance or electrostatic repulsion [46]. We should note that, in the absence of experimental validation (cell-based activation assays), the docking results would be hypothesis-generating without definitive evidence of functional divergence.

### 3.5. Absence of TLR1 in Another Transparent Fish: The X-Ray Tetra

**Figure 4:**
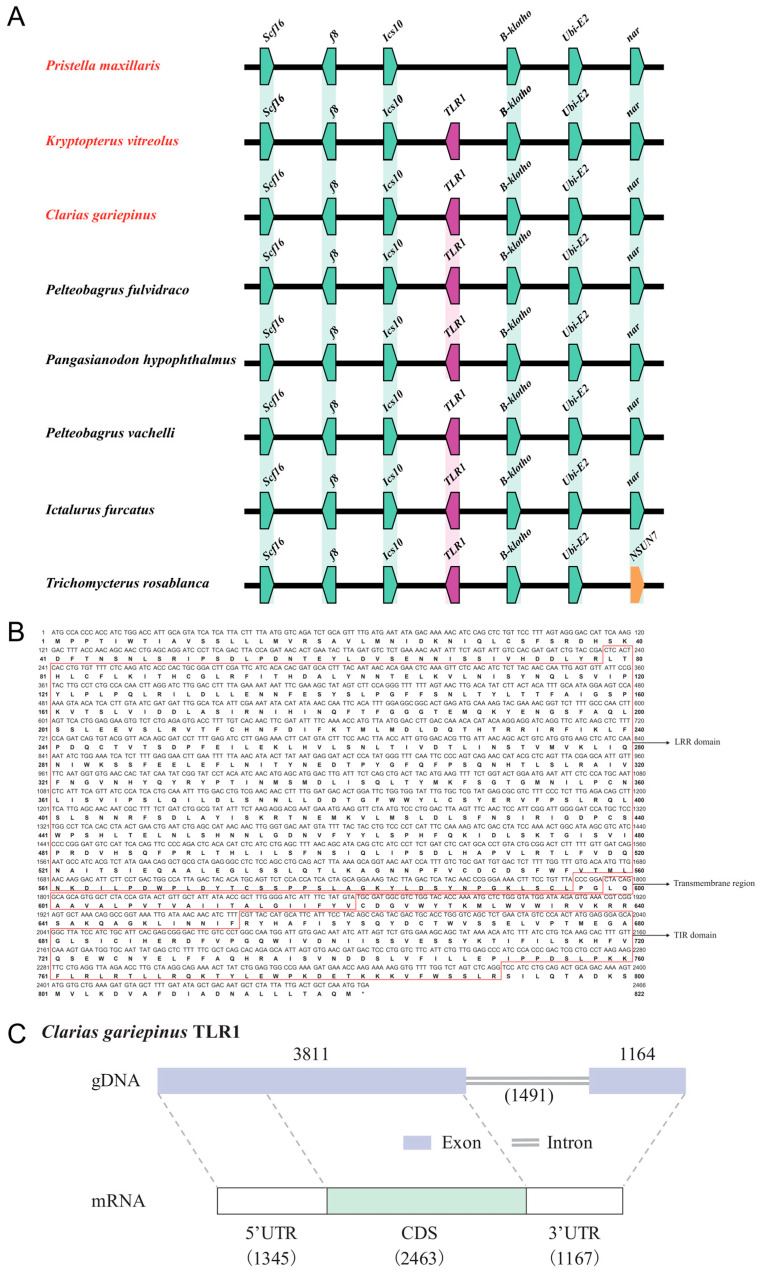
Absence of *TLR1* in another transparent fish, the X-ray tetra (*Pristella maxillaris*). (**A**) Conserved synteny (gene neighborhood) of *TLR1* across representative teleost genomes, showing the relative position of *TLR1* and flanking genes. (**B**) Nucleotide sequence and deduced amino acid sequence of *CgTLR1*, with annotated LRR domain, transmembrane region, and TIR domain. (**C**) Gene structure of *CgTLR1*, illustrating gDNA and mRNA organization, exon–intron architecture, and the lengths of the 5′ UTR, CDS, and 3′ UTR, respectively. “*” indicates a stop codon.

Synteny analysis revealed that the *TLR1* locus and its flanking paralogous gene blocks (containing *Ics10*, *f8*, *Scf16*, *B-klotho*, *Ubi-E*2, and *nar*) are highly conserved in order and orientation among six examined fish species, including *Cg*, *Kv*, *Pelteobagrus fulvidraco*, *Pangasianodon hypophthalmus*, *Pelteobagrus vachelli*, *Ictalurus furcatus*, and *Trichomycterus rosablanca*. However, in the corresponding genomic region of the X-ray tetra (*Pm*), although the same conserved neighboring gene blocks are present in an identical arrangement, the *TLR1* gene is absent within this block (Figure 4A).

The nucleotide sequence of the *CgTLR1* gene and its deduced amino acid sequence are provided (Figure 4B). The ORF is approximately 2466 bp in length and encodes a protein of 821 amino acids. The protein sequence can be clearly categorized into the typical structural features, including an N-terminal extracellular domain containing multiple LRRs (responsible for ligand recognition) and a single transmembrane region that anchors the receptor in the cell membrane, as well as a C-terminal intracellular TIR domain that interacts with downstream proteins and mediates signal transduction. The overall structure indicates that *Cg*TLR1 possesses the characteristic features of a transmembrane pattern-recognition receptor, providing a sequence basis for subsequent functional studies.

In Figure 4C, genomic and transcript structures of the *CgTLR1* gene are summarized. At the genomic DNA (gDNA) level, the gene consists of two exons (3811 and 1164 bp respectively) and one intron (1491 bp in length); at the mRNA level, the transcript contains a 5′UTR, a coding sequence (CDS), and a 3′UTR, with lengths of 1345, 2463, and 1167 bp, respectively.

## 4. Discussion

Among all examined species in this study, *TLR* genes other than *TLR21* were ubiquitously present (Table 2) and evolutionarily conserved (Figure 1), demonstrating their essential and non-redundant functions in both immunological and fundamental physiological processes of various siluriform fishes. Our current study, through comparative genomic analysis, has systematically revealed for the first time the unique evolutionary pattern of the *TLR* gene family in the glass catfish, characterized by “gene number reduction coupled with domain expansion”. That is to say, while consistently detected in seven siluriform species, it was specifically and completely absent in the glass catfish (Figure 2A), which confirms a lineage-specific gene loss event for *KvTLR21*.

From an evolutionary perspective, the absence of *KvTLR21*, a key pattern recognition receptor for microbial DNA detection [47], may represent an adaptive reorganization of the immune system during the evolution of the transparent glass catfish. This species may compensate for the loss by upregulating alternative receptors (such as genomic expansion of *TLR9*) or signaling pathways to maintain its pathogen defense capabilities [48,49]. The predicted binding energy (kcal/mol) from docking is typically a negative value, with a range between −5 and −9 that are generally considered indicative of stable binding. Within this range, any value close to −9 (with a larger absolute magnitude) usually imply stronger predicted binding and formation of a more stable complex [50].

The molecular docking results in this study showed significant sequence-specific differences in the binding stability of *CgTLR21* to different CpG ODNs (Figure 3), indicating that even in many species in which this gene is present, its function has become highly specialized. Specifically, the lowest binding energy of CpG-B 1681 suggests a more stable receptor–ligand complex, which may translate into more efficient CpG-DNA recognition and potentially stronger downstream activation of TLR21-mediated innate immune signaling.

By completely losing *TLR21*, the glass catfish may have fundamentally restructured its pathogen recognition network [51] to reduce the metabolic cost of immunity and adapt to its unique ecological niche. Meanwhile, this study identified absence of the *TLR1* gene in another transparent fish species, the X-ray tetra, within a conserved genomic block (Figure 4A). Interestingly, although binding to different ligands, both TLR21 and TLR1 activate the immune system through the same downstream MyD88-dependent signaling pathway [26,27,52,53]. In summary, these observations raise a working hypothesis that the evolution of transparent phenotypes may be associated with lineage-specific loss of certain *TLR* gene(s); however, alternative explanations, such as neutral gene loss, relaxed selection, or other ecological factors could also produce similar patterns. In the future, broader comparative genomic sampling across more species, tests of selective pressure (dN/dS), and expression and functional validation of key genes will help distinguish among these possibilities. This association may stem from fundamental changes in the immune microenvironment caused by the transparent body wall (such as increased UV exposure and higher rates of direct pathogen contact), thereby driving targeted remodeling of the immune recognition system. A transparent phenotype may increase internal light/UV penetration, which could influence skin and mucosal barrier integrity, microbiome composition, and exposure to environmental stressors and pathogens, thereby potentially imposing distinct selective pressures on innate immune sensing pathways. In this study, we observed lineage-specific loss of different *TLR* genes in various transparent fishes, such as *TLR21* in the glass catfish whereas *TLR1* in the X-ray tetra. These interesting findings suggest that transparent fishes may have experienced clade-specific remodeling of *TLR* repertoires, reflecting potential adaptation to altered ecological conditions.

From an evolutionary standpoint, gene loss is often regarded as an important adaptive evolutionary strategy [54]. The loss of *TLR21* in the glass catfish occurred after its divergence from its relative *Clarias gariepinus* (Figure 2D), and the high conservation of flanking gene synteny (Figure 2A) strongly supports that this was a secondary loss event in evolutionary history. Combined with the significant domain expansion observed in several TLR proteins (such as TLR5, 7, and 20) in this study (Figure 1), and based on the observed differences in the *TLR* gene copy number and domain composition, it is possible that this normal (non-transparent) reference species exhibits a distinct genomic configuration of the *TLR* repertoire; however, any functional optimization or increased functional complexity remains a hypothesis in the absence of empirical validation [55]. In our results, *KvTLR5*, *KvTLR7*, and *KvTLR20* show pronounced sequence/structural expansion, which may reflect functional adaptation to broaden or modify pathogen-recognition capacity in the absence of *TLR21*.

The lineage-specific evolutionary patterns of immune genes identified in this study extend beyond genomics into ecological and practical dimensions. Ecologically, the loss of key recognition genes such as *TLR21* may reflect adaptive responses to the specific habitats of the glass catfish, offering a model for understanding immune remodeling under environmental selection. From a societal perspective, as an important ornamental species, elucidating these immune adaptations can directly inform strategies to improve disease prevention and animal welfare in the aquarium trade, thereby reducing industry losses. Looking forward, the molecular mechanisms revealed here (such as CpG-ODN binding specificity) hold translational potential for predicting species responses to environmental change and emerging pathogens, and they also support rational design of immunostimulants or adjuvants for broader teleost aquaculture, which may contribute to the sustainable development of the fishery industry.

## 5. Conclusions

Through systematic genomics comparisons, synteny analysis, and molecular docking, this study has, for the first time, confirmed the specific loss of the *TLR21* gene in the transparent glass catfish. Our results revealed a unique evolutionary pattern in the *TLR* family, characterized by simultaneous gene loss (especially *KvTLR21*) and domain expansion (for TLR5, 7, and 20). Combined with the case of *TLR1* loss in another transparent fish species, the X-ray tetra, these results indicate that transparent fish may commonly utilize the loss of specific *TLR* gene(s) as an important way for adaptive evolution of the immune system, a process likely driven by their unique physiological traits (such as transparency). The practical role of gene loss, such as *TLR21* in *Kv* or *TLR1* in *Pm*, requires further investigation. Future studies may functionally validate these findings by using CRISPR/Cas9-mediated loss-of-function experiments targeting *TLR21* and other retained *TLR*s, as well as by assessing downstream immune responses to CpG ODN stimulation and pathogen challenge. These interesting findings provide a key candidate for improving our understanding of the adaptive evolution of vertebrate immune gene families and lay a theoretical foundation for the in-depth exploration of the molecular links between physiological specialization and immune adaptation in transparent vertebrates, including fish.

## Figures and Tables

**Table 1:** Genetic resources of the downloaded genome assemblies and *TLR* genes.

**Species Name**	Data Acquisition Link	Accession Number
*Clarias fuscus*	https://figshare.com/articles/dataset/Genome_assembly_and_annotation_information_of_female_i_Clarias_fuscus_i_/26968489, (accessed on 20 July 2024)	GCA_046453815.1
*C. gariepinus*	https://ftp.ncbi.nlm.nih.gov/genomes/all/GCF/024/256/425/GCF_024256425.1_CGAR_prim_01v2/, (accessed on 20 July 2024)	GCA_024256425.2
*K. vitreolus*	https://figshare.com/articles/dataset/_b_A_telomere-to-telomere_chromosome-level_genome_of_glass_catfish_b_b_i_Kryptopterus_vitreolus_i_b_/28333385?file=52098524, (accessed on 20 July 2024)	GCA_044706155.1
*Ictalurus furcatus*	https://ftp.ncbi.nlm.nih.gov/genomes/all/GCF/023/375/685/GCF_023375685.1_Billie_1.0/, (accessed on 20 July 2024)	GCA_023375685.2
*Pristella maxillaris*	https://figshare.com/articles/dataset/The_genome_and_annotation_of_i_Pristella_maxillaris_i_/27901167?file=50813691, (accessed on 20 July 2024)	GCA_045781885.1
*Pangasianodon hypophthalmus*	https://ftp.ncbi.nlm.nih.gov/genomes/all/GCF/027/358/585/GCF_027358585.1_fPanHyp1.pri/, (accessed on 20 July 2024)	GCA_027358585.1
*Pelteobagrus vachelli*	https://www.ncbi.nlm.nih.gov/datasets/genome/GCF_030014155.1/, (accessed on 20 July 2024)	GCF_030014155.1
*Tachysurus fulvidraco*	https://ftp.ncbi.nlm.nih.gov/genomes/all/GCF/022/655/615/GCF_022655615.1_HZAU_PFXX_2.0/, (accessed on 20 July 2024)	GCF_003724035.1
*Trichomycterus rosablanca*	https://ftp.ncbi.nlm.nih.gov/genomes/all/GCF/030/014/385/GCF_030014385.1_fTriRos1.hap1/, (accessed on 20 July 2024)	GCA_030014385.1

**Table 2:** Genome-wide identification of *TLR* genes in the assembled genome of the glass catfish (*Kv*) and its relative, the North African catfish (*Cg*).

Gene Name	Full Length (bp) *	ORF (bp) **	Protein Accession Number	Deduced Protein (aa)	Molecular Weight (kDa)	Genomic Position
*Cg* *TLR 1*	6466	2466	XP_053364231.1	821	93.9	NC_071111.1: 12160120-12166586
*Cg* *TLR 2*	6332	2394	XP_053332572.1	797	92.4	NC_071117.1: 29015012-29021344
*CgTLR 3*	8918	2709	XP_053336254.1	902	103.6	NC_071119.1: 3523259-3532177
*CgTLR 5*	9885	2469	XP_053370797.1	822	101.1	NC_071115.1: 4460271-4470156
*CgTLR 7*	3967	3198	XP_053353400.1	1065	122.8	NC_071104.1: 1013192-1017159
*CgTLR 8*	7951	3183	XP_053353401.1	1060	123.3	NC_071104.1: 1004099-1012050
*CgTLR 9*	7501	3204	XP_053360697.1	1067	122.6	NC_071108.1: 24685621-24693122
*CgTLR 13*	7019	2973	XP_053349846.1	990	114.1	NC_071103.1: 10385652-10392671
*CgTLR 18*	5623	2547	XP_053350949.1	848	97.8	NC_071103.1: 18566052-18571675
*CgTLR 20*	3199	3081	XP_053358352.1	1036	117.8	NC_071107.1: 35684746-35687945
*CgTLR 21*	3011	2946	XP_053350396.1	981	113.4	NC_071103.1: 13158780-13161791
*KvTLR1*	5913	2487	augustus-scaffold_3-processed-gene-19.39-mRNA-1	828	94.7	scaffold_3:5864176-5870089
*KvTLR2*	9670	2403	augustus-scaffold_22-processed-gene-1.25-mRNA-1	800	92.0	scaffold_22:438534-448204
*KvTLR3*	5444	2694	maker-scaffold_16-augustus-gene-63.31-mRNA-1	897	102.0	scaffold_16:19107590-19113034
*KvTLR5*	30,855	7032	augustus-scaffold_12-processed-gene-67.15-mRNA-1	2343	264.9	scaffold_12:20183769-20214654
*KvTLR7*	25,855	8322	augustus-scaffold_6-processed-gene-88.9-mRNA-1	2773	313.7	scaffold_6:26546654-26572509
*KvTLR9*	11,650	1572	augustus-scaffold_1-processed-gene-32.2-mRNA-1	523	59.4	scaffold_1:9564569-9576219
*KvTLR13*	2882	2883	augustus-scaffold_27-processed-gene-40.16-mRNA-1	960	108.4	scaffold_27:12131308-12134190
*KvTLR20*	32,561	4299	augustus-scaffold_29-processed-gene-7.0-mRNA-1	1432	163.5	scaffold_29:2071883-2104444

* Full length (bp) refers to the full-length gene sequence including untranslated regions (UTRs), whereas ** ORF (bp) indicates the coding sequence (CDS) only.

## Data Availability

The original contributions presented in this study are included in the article. Further inquiries can be directed to the corresponding authors.
